# Retinoic acid receptor β modulates mechanosensing and invasion in pancreatic cancer cells via myosin light chain 2

**DOI:** 10.1038/s41389-023-00467-1

**Published:** 2023-05-02

**Authors:** Carlos Matellan, Dariusz Lachowski, Ernesto Cortes, Kai Ning Chiam, Aleksandar Krstic, Stephen D. Thorpe, Armando E. del Río Hernández

**Affiliations:** 1 Cellular and Molecular Biomechanics Laboratory, Department of Bioengineering, Imperial College London, London, SW7 2AZ UK; 2 Department of Physiology, School of Medicine, Autonomous University of Madrid, 28029 Madrid, Spain; 3 UCD School of Medicine, University College Dublin, Dublin, Ireland; 4 Systems Biology Ireland, University College Dublin, Dublin, Ireland; 5 UCD Conway Institute of Biomolecular & Biomedical Research, University College Dublin, Dublin, Ireland; 6 Trinity Centre for Biomedical Engineering, Trinity College Dublin, Dublin, Ireland

**Keywords:** Pancreatic cancer, Cancer microenvironment, Cytoskeleton

## Abstract

Pancreatic ductal adenocarcinoma (PDAC) is the most common and lethal form of pancreatic cancer, characterised by stromal remodelling, elevated matrix stiffness and high metastatic rate. Retinoids, compounds derived from vitamin A, have a history of clinical use in cancer for their anti-proliferative and differentiation effects, and more recently have been explored as anti-stromal therapies in PDAC for their ability to induce mechanical quiescence in cancer associated fibroblasts. Here, we demonstrate that retinoic acid receptor β (RAR-β) transcriptionally represses myosin light chain 2 (MLC-2) expression in pancreatic cancer cells. As a key regulatory component of the contractile actomyosin machinery, MLC-2 downregulation results in decreased cytoskeletal stiffness and traction force generation, impaired response to mechanical stimuli via mechanosensing and reduced ability to invade through the basement membrane. This work highlights the potential of retinoids to target the mechanical drivers of pancreatic cancer.

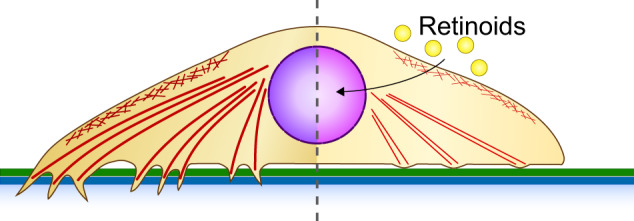

## Introduction

Pancreatic ductal adenocarcinoma (PDAC), the most common (90%) form of pancreatic cancer [1], is a highly aggressive malignancy, being the 3rd leading cause of cancer related death in the US [2] and the 7th worldwide [3]. The 5-year survival rate remains low (~10%) due to the fast progression of the disease, its metastatic potential and the difficult diagnosis [2]. This dismal prognosis and the ineffectiveness of classical treatments calls for novel therapeutic strategies to tackle the burden of pancreatic cancer.

In the last decade, the biomechanical interaction between the cancer cell and the tumour microenvironment has gained interest as a factor driving the onset and progression of cancer [4]. From a biomechanical standpoint, PDAC is characterised by a highly stiff and fibrotic tumour microenvironment. This aberrant, collagen-rich stroma is populated by activated pancreatic stellate cells (PSCs), which remodel the extracellular matrix (ECM) into a cancer-permissive microenvironment [5, 6]. This microenvironment, in turn, promotes epithelial-to-mesenchymal transition [7, 8], guides cancer cell migration [9–11], increases secretion of matrix metalloproteinases [12–14], promotes chemoresistance [15], and its stiffness correlates with metastatic potential and response to treatment [7, 16]. Bidirectional crosstalk between tumour cells and activated PSCs maintains the tumour microenvironment, sustains PSC activation and drives cancer cell malignancy [17].

The retinoic acid receptors RAR-α, RAR-β and RAR-γ are the three members of the retinoic acid receptor (RAR) subfamily of nuclear receptors, which play a wide variety of roles in embryonic development, morphogenesis and proliferation [18]. RARs bind their transcription partners, retinoid X receptors (RXRs), to form heterodimeric transcription factors that bind specific sites on target genes known as retinoid acid response elements (RAREs) [19]. RAR/RXR dimers are activated by the binding of retinoids, the active forms of vitamin A, which triggers the transcriptional activation or repression of target genes. Of the three family members, RAR-α is ubiquitously expressed, while RAR-β and RAR-γ are tissue specific [20]. Interestingly, the expression of RAR-β is lost or downregulated in a variety of carcinomas, including breast [21, 22], lung [23, 24], liver [25] and pancreatic [26, 27] cancer among others. This dysregulation in the expression of RAR-β often accompanies the early stages of cancer and may be concomitant with its development [28].

Retinoids have been explored as a treatment for cancer in diverse contexts. Retinoic acid treatment reduces proliferation and tumour growth in a variety of cancers, including lung, breast, oral and skin cancer [22, 29, 30], and all-trans retinoid acid (ATRA) is currently used as treatment for acute promyelocytic leukaemia (APL) [31]. Retinoid signalling also downregulates serum response factor (SRF)-dependent genes [32], which include several cytoskeletal proteins [33], connective tissue growth factor [34], and several microRNAs [35]. While its anti-proliferative effect is well characterised, our group recently demonstrated that RAR-β activation via ATRA can regulate the mechanical activity of cancer associated fibroblasts (CAFs) [11, 25]. These findings position RAR-β as an important player in cancer mechanobiology and an attractive target for cancer therapy. However, the mechanism by which RAR-β modulates the mechanical activity of cancer cells remains unexplored.

Here, we investigate the mechanism of mechano-regulation by retinoids in pancreatic cancer cells. First, we demonstrate that the expression of RAR-β is downregulated in PDAC tissues, a loss that correlates with tumour stage and is concomitant with an increase in myosin light chain 2 (MLC-2) expression, but can be restored via RAR-β activation. We further explored this mechanism and found that RAR-β activation transcriptionally downregulates MLC-2, the core regulatory component of the contractile actomyosin machinery. Using elastic micropillar arrays, magnetic tweezers and atomic force microscopy, we demonstrate that RAR-β-dependent MLC-2 repression decreases the mechanical activity of PDAC cells including traction force generation and mechanosensing, reduces the stiffness of cancer cells, and impairs their ability to invade through the basement membrane. Together, our results shed new light into the potential of retinoids as mechano-modulating drugs in pancreatic cancer.

## Materials and methods

### Cell culture and reagents

Suit2-007 cells (metastatic PDAC cell line) were kindly donated by Prof. Malte Buchholz from Philipps-Universität Marburg. Suit2 cells were cultured in Dulbecco’s Modified Eagle’s Media (Merck, Dorset, UK, D8437) supplemented with 10% v/v foetal bovine serum (FBS; Merck, F7524), 2 mM l-glutamine (Merck, G7513), 1% v/v penicillin/streptomycin (Merck, P4333) and 1% v/v fungizone Amphotericin B (Gibco, Thermo Fisher Scientific, UK, 15290-026). Cells were incubated at 37 °C, with 5% CO_2_. For all RAR-β agonist treatments, cells were exposed to 1 μM RAR-β agonist (CD 2314, Tocris, Abingdon, UK, 3824) 24 h prior to experiments. Gene transfection was performed 48 h prior to experiments, utilising the Neon transfection system (Thermo Fisher Scientific) with 2 μg MLC-2 plasmid (pEGFP-MRLC1, a gift from Tom Egelhoff, Addgene, #35680), 10 μg RAR-β siRNA (Santa Cruz, SC-29466) or 10 μg control siRNA (siRNA-Scr, Santa Cruz, SC-37007). Tissue micro arrays (TMAs) were obtained from Biomax (RAR-β: PA803, MLC-2: PA242e). Primary antibodies used were rabbit anti-RAR-β (Abcam, Cambridge, UK, ab53161), rabbit anti-MLC-2 (Cell Signaling Technology, 3672S), mouse anti-PAN Cytokeratin (Abcam, ab6401), rabbit anti-YAP (Cell Signaling Technology, 4912S) and rabbit anti-laminin (Sigma-Aldrich, L9393). See Supplementary Methods for immunostaining, tissue microarray, ChIP-seq and RT qPCR details.

### Elastic micropillar arrays

Elastic micropillar arrays were fabricated in polydimethylsiloxane (PDMS). PDMS (Sylgard 184, Dow) was mixed in a 1:10 weight ratio according to the manufacturer specifications, poured on a silicon mould and cured at 60 °C for 1 h, resulting in PDMS with a spring constant *k* = 1.36 nN/µm. After curing, PDMS pillars were peeled-off the mould in phosphate buffered saline (PBS) and stored at 4 °C. Prior to seeding cells, PDMS pillars were coated with 10 μl/mL fibronectin (FN; Gibco, PHE0023) in PBS for 1 h at 37 °C. Cells were seeded on the FN-coated pillars and incubated for 1 h at 37 °C and 5% CO_2_ before analysing them. Each sample was imaged at 37 °C for up to 30 min on an inverted microscope (Nikon Ti Eclipse, C-LHGFI HG Lamp, CFI Plan Fluor 40× NA 0.6 air objective) fitted with a Neo sCMOS camera (Andor, Oxford, UK) using NIS elements AR software. Each cell was recorded for 1 min with a frame rate of 1 frame/s. Data analysis was carried out with a custom MATLAB script to quantify pillar deflection and traction forces exerted on each pillar were calculated based on the deflection of the pillar and the spring constant.

### Magnetic tweezers

Magnetic beads (4.5 µm, Dynabeads M-450, Thermo Fisher Scientific) were coated with fibronectin (Gibco, PHE0023) following the manufacturer’s instructions. Suit2 cells were incubated with fibronectin-coated beads for 30 min at 37 °C and then thoroughly washed with PBS to remove unbound beads. Individual cell-bound beads were then subjected to a pulsatile force regime using magnetic tweezers consisting of a 3 s, 6 nN force pulse, followed by a 4 s rest period, repeated for 12 pulses over ~100 s. The bead trajectories were recorded using an inverted microscope (Nikon Ti-Eclipse, C-LHGFI HG Lamp, CFI Plan Fluor 40× NA 0.6 air objective) fitted with a Neo sCMOS camera (Andor) with NIS elements AR software, and analysed using a custom MATLAB script. The amplitudes of each pulse were extracted from bead trajectories and normalised to the 1st pulse. The amplitudes of the 1st and 12th pulse were compared to quantify the decrease in amplitude of the bead movement as a result of cytoskeletal reinforcement.

### Atomic force microscopy

Cell stiffness was analysed with AFM nanoindentation using a Nanowizard 4 (Bruker, Coventry, UK) in contact – force spectroscopy mode. Nanoindentation measurements were carried out with an MLCT silicon nitride probe (Bruker) with a nominal spring constant of 0.03 nN/m with a 15 µm polystyrene bead attached to the tip. Prior to cell analysis, the sensitivity of the probe was calibrated by measuring the force–distance slope in the AFM software on an empty petri dish region. Nanoindentation of individual cells was conducted at 5 µm/s to a set point of 1 V (~1 nN force set point). Cells were indented at a point between the nucleus and the cell periphery to characterise the cytoskeletal stiffness. The stiffness (Young’s modulus) of individual cells was calculated from the force-distance curves using the AFM software with the Hertz model [36].

### Mesentery isolation and invasion assay

Mesenteries were isolated and prepared as described previously [37]. Briefly, 1.5 mL Eppendorf tubes were cut to create a tube of constant diameter approximately 1 cm in height to be used as a frame for the mesenteries. Mesenteries were isolated from mice intestines (generously provided by Dr Charlotte Dean from Imperial College London) using Vetbond tissue adhesive (3 M, 1469SB). Mesenteries were immediately incubated for 1 h in sodium azide (NaN_3_, Merck, S2002) diluted in PBS for preservation, and decellularised by 1 h incubation with 1 M ammonium hydroxide solution (NH_4_OH, Merck, 09859). Mesenteries were then washed and stored in Dulbecco’s PBS (D-PBS; Merck, D8537) at 4 °C. Cells were collected using 0.25% trypsin-EDTA solution (Merck, T4049), centrifuged, resuspended in serum-free media, and seeded inside the Eppendorf ring on top of mesenteries that were previously placed in wells with media containing 10% FBS to drive the invasion assay. Mesenteries were transferred to a new culture dish every 24 h.

Mesenteries were fixed on days 3 and 5 with 4% paraformaldehyde for 10 min. Invasion was assessed using confocal fluorescence microscopy (Ti Eclipse, Nikon). For each mesentery, an average of 5 randomly selected fields of view were analysed, with an average of 10 cells per field of view. Percentage cell invasion was quantified from cross-sectional confocal images as the ratio of the height of the cell below the mesentery bilayer to the total cell height. For cumulative invasion, after the mesenteries were transferred to a new well, the number of cells that had invaded thought the mesentery and attached to the bottom of the wells were quantified within randomly selected regions of interest (ROI) by imaging on a bright field inverted microscope (Motic, AE31 trinocular). The number of cells per mesentery per ROI were quantified every 24 h, and cumulated over a period of 5 days.

### Statistical analysis

Statistical analyses were conducted using GraphPad Prism 8 (GraphPad). Data were collected from multiple repeats of replicate biological experiments. Data were tested for normality prior to analysis using two-sided t-test or analysis of variance (ANOVA) with Tukey’s or Dunnet’s pairwise comparisons for data adhering to a normal distribution. Non-parametric datasets were analysed using the Mann–Whitney test, or Kruskall–Wallis test with Dunn’s pairwise comparisons. The Brown–Forsythe and Welch correction was used for ANOVA tests with unequal variances. Significance was set at *P* < 0.05, and *P* values were adjusted for multiple comparisons where appropriate.

## Results

### RAR-β expression is reduced in pancreatic ductal adenocarcinoma

**Fig. 1:**
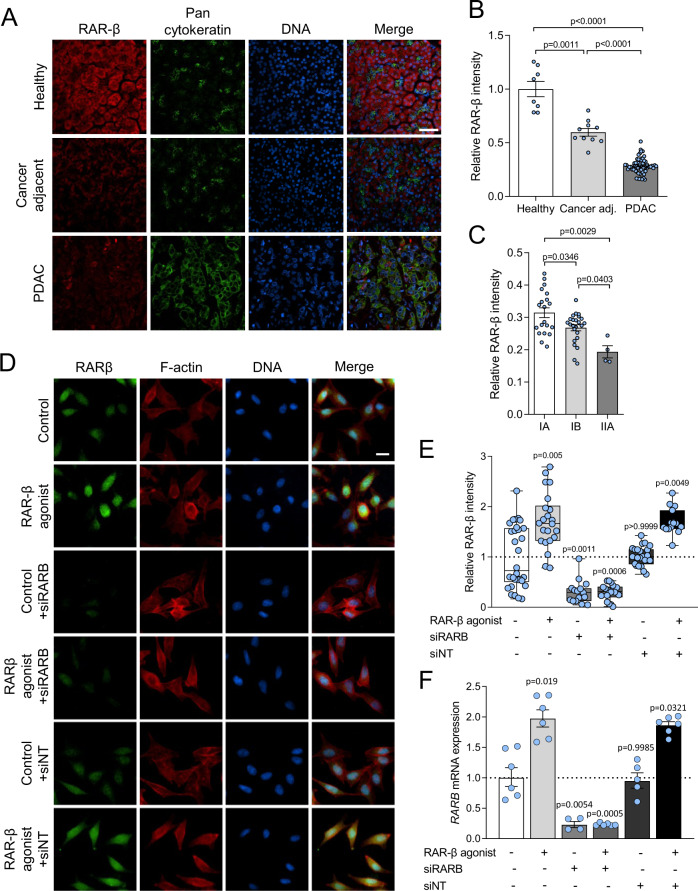
Expression of RAR-β in tissue arrays. **A** Representative immunofluorescent images for RAR-β (red), PAN cytokeratin (green) and DNA (blue) in healthy pancreas, cancer adjacent tissue and PDAC tissue microarrays. Scale bar: 50 µm. **B** Quantification of the immunofluorescent staining in (A). Mean ± s.e.m., *n* = 8, 10 and 60 for healthy, cancer adjacent and PDAC tissue microarrays, respectively. Brown-Forsythe and Welch ANOVA tests with Tukey’s post-hoc test. **C** Quantification of the mean fluorescence intensity for RAR-β on tissue micro arrays for stage IA, IB and IIA PDAC relative to healthy pancreatic tissue. Mean ± s.e.m., *n* = 20, 24 and 4 for stages IA, IB, and IIA, respectively. One way ANOVA test with Tukey’s post-hoc test. **D** Immunofluorescence analysis of RAR-β expression at the protein level. Representative images for control, RAR-β agonist, *RARB* siRNA (siRARB), RAR-β agonist + siRARB, non-targeting siRNA (siNT), and RAR-β agonist + siNT, respectively. Scale bar: 20 µm. **E** Quantification of immunofluorescent staining in (D). Mean ± s.e.m., *n* = 30, 22, 15, 18, 22 and 15, respectively. Kruskal–Wallis test with Dunn’s post-hoc test. **F** Relative expression of RAR-β in control, RAR-β agonist, RAR-β siRNA, RAR-β agonist + RAR-β siRNA, non-targeting siRNA (siNT) and RAR-β agonist + siNT, respectively, as measured by mRNA RT qPCR normalised to RPLP0. Mean ± s.e.m., *n* = 6, 6, 4, 6, 5 and 6. *P*-values indicate significant difference relative to control by Brown-Forsythe and Welch one way ANOVA tests with Dunnett’s post-hoc test.

Retinoic acid receptor β (RAR-β) has been postulated to act as a tumour suppressor [38, 39]. Its loss is associated with poor prognosis in colorectal cancer [40], and its expression is dysregulated or supressed in several types of cancer including lung, cervix and breast cancer [20]. Here, we analysed RAR-β expression in pancreatic ductal adenocarcinoma (PDAC) and healthy tissue microarrays (TMAs). Healthy tissues exhibited high expression of RAR-β (Fig. 1A, B) but this expression was significantly reduced in PDAC and PDAC-adjacent tissues, with a ~ 70% reduction in expression between healthy and PDAC tissue. RAR-β expression in PDAC was further localised to areas of low PAN-cytokeratin expression, a marker of pancreatic cancer cells, which was abundantly expressed in PDAC tissue but negligible in healthy pancreatic tissue. Analysis of cancer adjacent tissues indicated a similar trend, with increased PAN-cytokeratin expression and decreased RAR-β expression (~50% compared to healthy tissues). Similarly, we observed a correlation between the loss of RAR-β expression and the tumour stage (Fig. 1C), with a significant reduction in RAR-β expression between IA, IB and IIA tumour tissues. Together, these results suggest that the loss of RAR-β expression is associated with PDAC progression and the development of the malignant phenotype, consistent with previous reports [38, 41].

Positive RAR-β autoregulation, i.e., an increase in RAR-β expression upon retinoid treatment, has been previously reported in humans, mice, and rats [42–45]. Here, we hypothesised that treatment with retinoids could restore RAR-β signalling in pancreatic cancer cells. To this end, we treated Suit2 cells, a malignant PDAC cell line, with the selective RAR-β agonist CD 2314 for 24 h. Immunofluorescence analysis of RAR-β expression in Suit2 cells revealed a nearly 2-fold increase in the expression of RAR-β at the protein level upon treatment with RAR-β agonist (Fig. 1D, E). Analysis of *RARB* mRNA expression via qPCR revealed a similar increase in RAR-β agonist-treated cells compared to control (untreated) cells (Fig. 1F). Knock down of *RARB* via siRNA effectively decreased RAR-β expression at the protein and mRNA levels both in control cells and RAR-β agonist-treated cells, thus abrogating the effect of retinoids in RAR-β autoregulation. These results indicate that RAR-β autoregulation by retinoids can restore retinoid signalling in PDAC cells.

### RAR-β regulates MLC-2 transcription

When activated by retinoids, RAR-β forms a heterodimeric (RAR-β/RXR) transcription complex, and binds to retinoid acid response elements (RAREs) on target genes to regulate their expression. Retinoids have a pleiotropic effect on a variety of cellular programmes, from proliferation and lipid metabolism [46] to the regulation of immune system [47] or the cytoskeleton [32]. Our group has previously investigated the effect of ATRA as a mechano-modulator in cancer associated fibroblasts [11, 25].

**Fig. 2:**
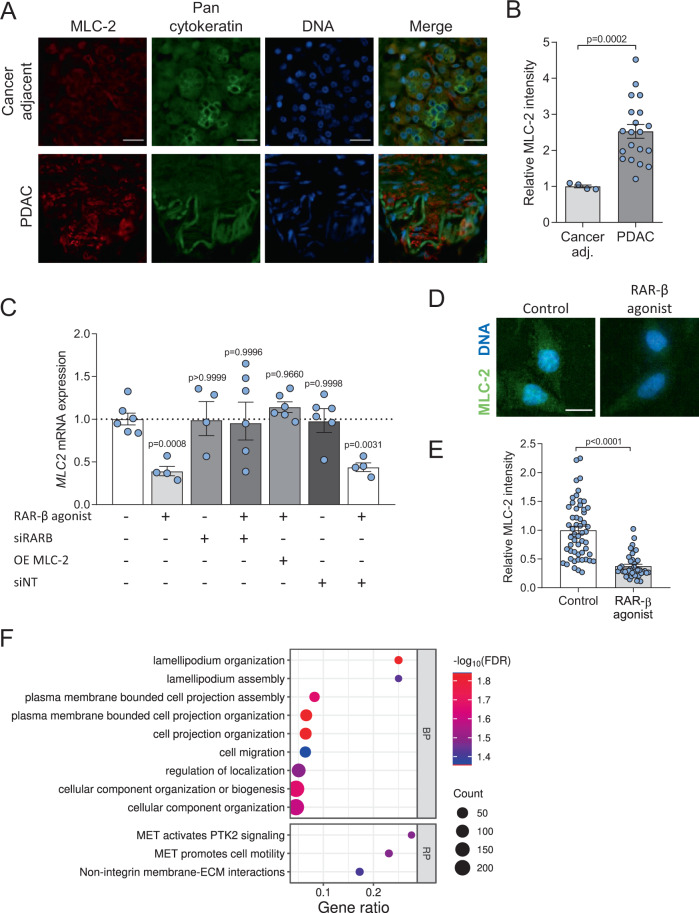
RAR-β activation downregulates expression and activity of MLC-2. **A** Representative immunofluorescent images for MLC-2 (red), PAN Cytokeratin (green) and DNA (blue) on cancer adjacent and PDAC tissue microarrays. Scale bar: 50 µm. **B** Quantification of MLC-2 immunofluorescent staining. Mean ± s.e.m., *n* = 4 and 20 for cancer adjacent and PDAC tissue microarrays, respectively. Mann–Whitney test. **C** Expression levels of *MLC2* quantified via RT qPCR normalised to RPLP0 and relative to control. Control, RAR-β agonist, *RARB* siRNA (siRARB), RAR-β agonist + siRARB, RAR-β agonist + MLC-2 overexpression (OE MLC2), non-targeting siRNA (siNT), and RAR-β agonist + siNT; geometric mean ± s.e.m., *n* = 6, 4, 4, 6, 6, 6 and 4, respectively. *P*-values denote significant difference relative to control by one way ANOVA test with Dunnett’s post-hoc test. **D** Representative images for MLC-2 (green) and DNA (blue) for control and RAR-β agonist. Scale bar: 20 µm. **E** Quantification of MLC-2 fluorescent staining in **D**. Mean ± s.e.m., *n* = 54 and 40 for control and RAR-β agonist, respectively. Mann–Whitney test. **F** Bubble plot presentation of gene ontology terms for biological process (BP) and Reactome pathways (RP) associated with RAR-β enriched genes. Terms were identified in STRING from ChIP-seq analysis of RAR-β agonist-treated Suit2 cells and are listed in order of gene ratio (enriched genes/genes in pathway) with colour representing -log_10_(false discovery rate) and dot size relating to the number of enriched genes in the pathway.

MLC-2 is a critical regulatory component of the actomyosin machinery. Phosphorylation of MLC-2 modulates force generation by non-muscle myosin II, the primary contractile apparatus in cancer cells, and is therefore associated with the regulation of cancer cell migration, invasion and mechanosensing. MLC-2 is upregulated in different cancer types, including melanoma [48], hepatocellular carcinoma [25], oesophageal squamous cell carcinoma [49], and PDAC [50], making it an important prognosis and therapeutic target in cancer biomechanics [51]. Transcription factor binding site analysis identified putative RAREs on multiple MLC-2 isoforms (Supplementary Fig. S1). Analysis of MLC-2 expression in normal cancer adjacent and PDAC tissue microarrays (TMAs) confirmed a 2.5-fold increase in MLC-2 expression in PDAC (Fig. 2A, B).

To characterise the effect of retinoid signalling on MLC-2, we analysed *MLC2* expression in Suit2 cells via RT qPCR and found that treatment with RAR-β agonist for 24 h significantly reduces the expression of *MLC2* at the mRNA level (Fig. 2C). This transcriptional downregulation was further confirmed in a second PDAC cell line, MIA PaCa-2 (Supplementary Fig. S2). On the other hand, RAR-β siRNA inhibited the effect of the agonist and restored control levels of *MLC2* expression, indicating that the downregulation of *MLC2* expression is RAR-β dependent. Likewise, transfection with a plasmid overexpressing *MLC2* restored the expression of *MLC2* even in the presence of RAR-β agonist, whereas it had no effect on the expression of RAR-β (Supplementary Fig. S3). Immunofluorescence analysis of MLC-2 expression at the protein level revealed a similar trend (Fig. 2D, E), with a significant reduction in MLC-2 upon treatment with RAR-β agonist. Collectively, these results indicate that retinoids transcriptionally downregulate MLC-2 expression via RAR-β.

To understand the mechanism through which RAR-β agonist drives MLC-2 transcription, we conducted chromatin immunoprecipitation sequencing (ChIP-seq) for RAR-β on Suit2 cells treated with RAR-β agonist. Suit2 cells treated with RAR-β antagonist provided a control condition. Peak calling identified 3244 regions enriched for RAR-β which were associated with 2148 genes. 29.9% of these sites were upstream of the transcription start site (TSS). While RAREs were identified on MLC-2 isoforms MLY-2, MYL-9 and MYL-10 (Supplementary Fig. S1), we did not observe RAR-β binding to these sites in response to agonist treatment. Analysis of RAR-β enriched genes using STRING [52] identified nine significant biological process gene ontology terms and three Reactome pathways all of which involve cytoskeletal remodelling (Fig. 2F and Supplementary Fig. S4A). Ingenuity Pathway Analysis conducted on RAR-β enriched genes identified 189 significantly altered canonical pathways which include RXR and RAR activation in addition to several adhesion and contractility related pathways (Supplementary Fig. S4B).

Given the importance of MLC-2 in regulating the biomechanical activity of cancer cells, we decided to analyse the effect of RAR-β activation on the expression of YAP-1, a well-known marker of mechanically active cancer cells. Treatment with RAR-β agonist for 24 h decreased the nuclear accumulation of YAP-1 (i.e., its activation) (Supplementary Fig. S5), an effect that is abrogated when the receptor is knocked down (siRNA). These results point towards a RAR-β-dependent mechanism of mechano-modulation and prompted us to investigate the downstream effects of MLC-2 downregulation on the mechanical activity of PDAC cells.

### RAR-β activation inhibits traction force generation, cytoskeletal stiffness and mechanosensing

MLC-2 is a fundamental regulator of actomyosin organisation and contractility, which governs the cell’s ability to generate forces and to mechanically interact with their microenvironment. A dynamic and functional actomyosin machinery is critical for cancer cells to migrate and invade other tissues, to respond to mechanical cues and to remodel their microenvironment. Based on the previous finding that RAR-β transcriptionally downregulates MLC-2, we decided to assess the effect of RAR-β activation on the mechanical activity of PDAC cells, including contractility, mechanosensing and cytoskeletal stiffness.

**Fig. 3:**
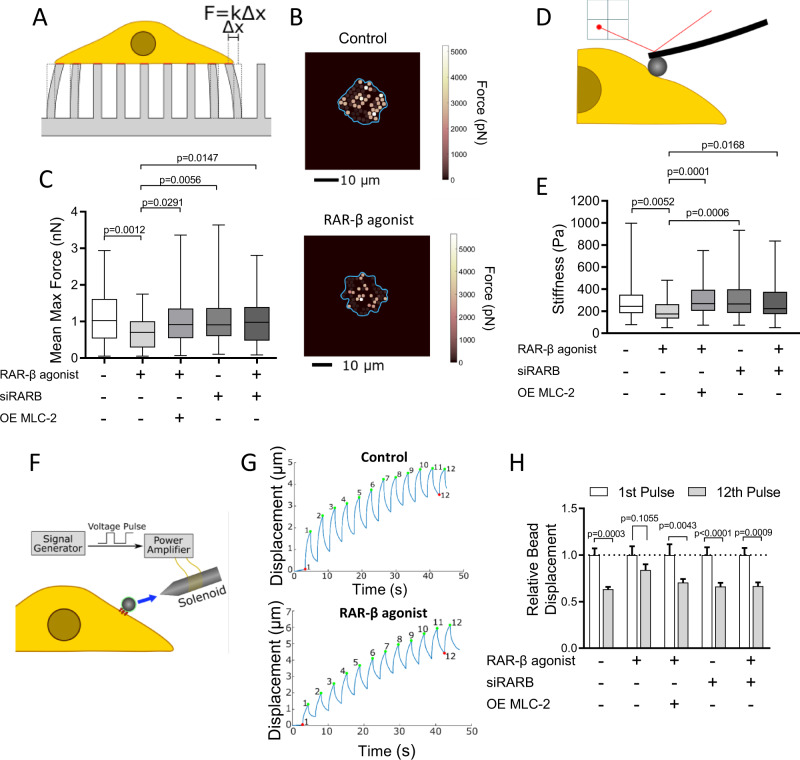
RAR-β activation impairs traction force generation, cytoskeletal stiffness and mechanosensing in pancreatic cancer cells. **A** Schematic of the elastic micropillar array setup to quantify cellular traction forces. **B** Heat map of the traction force distribution in control and RAR-β agonist-treated Suit2 cells. The cell body is outlined in blue. Scale bar: 10 µm. **C** Quantification of mean maximum traction force exerted by Suit2 cells on elastic pillars for Control, RAR-β agonist, RAR-β siRNA (siRARB), RAR-β agonist + siRARB, and RAR-β agonist + MLC-2 overexpression (OE MLC-2). Mean ± s.e.m., *n* = 70, 53, 97, 110 and 87 cells, respectively. *P*-values indicate difference relative to RAR-β agonist-treated cells by Kruskal–Wallis test with Dunn’s post-hoc test. **D** Schematic of the AFM nanoindentation method to measure cell stiffness. **E** Cytoskeletal stiffness measured with AFM using a 15 μm bead and fitted to the Hertz model for control, RAR-β agonist, RAR-β siRNA (siRARB), RAR-β agonist + siRARB, and RAR-β agonist + MLC-2 overexpression (OE MLC-2). Mean ± s.e.m., *n* = 91, 58, 58, 56 and 64 cells, respectively. *P*-values indicate difference relative to RAR-β agonist-treated cells by Kruskal–Wallis test with Dunn’s post-hoc test, *p < 0.05, **p < 0.01, ***p < 0.001. **F** Schematic representation of the magnetic tweezers protocol used to measure mechanosensing in pancreatic cancer cells with the blue arrow indicating the magnetic pull applied to a fibronectin-coated bead on the cell surface. **G** Representative bead trajectories under the pulsatile force regime (12 force pulses) in control and RAR-β agonist-treated cells. A decrease in the displacement amplitude over the 12 pulses can be observed in control cells but not in RAR-β agonist-treated cells. **H** Relative bead displacement for the 1st and 12th pulses for control, RAR-β agonist, RAR-β siRNA (siRARB), RAR-β agonist + siRARB, and RAR-β agonist + MLC-2 overexpression (OE MLC-2). A significant difference between the amplitude of the 1st pulse and the 12th pulse is an indicator of mechanosensing as the cell reinforces in response to the applied force. Mean ± s.e.m., *n* = 41, 22, 28, 30 and 21 cells, respectively. Wilcoxon signed-rank test.

First, we investigated the effect of RAR-β on traction force generation using a previously established elastic micropillar platform. Micropillar arrays were fabricated from poly(dimethylsiloxane) (PDMS) via replica moulding and coated with fibronectin (FN) prior to cell seeding to enable cell attachment. Pillar displacements induced by cell-generated forces were monitored and converted to traction force maps used to quantify contractility (Fig. 3A, B). Control Suit2 cells generated a mean maximum traction force of 1.1 ± 0.1 nN (mean ± SEM, *n* = 70 cells), comparable to other PDAC cells [53], but their contractility was significantly reduced (0.7 ± 0.1, mean ± SEM, *n* = 53 cells) upon treatment with RAR-β agonist (72 h), consistent with the downregulation of MLC-2 expression (Fig. 3C). In contrast, knockdown of the receptor via RAR-β siRNA inhibited the effect of the agonist, resulting in a traction force similar to control, while overexpression of MLC-2 via transfection reversed the effect of RAR-β treatment and rescued control levels of traction force generation (1.0 ± 0.1 nN, mean ± SEM, *n* = 87 cells). These results were confirmed in a second PDAC cell line (MIA PaCa-2), with a ~ 50% reduction in mean maximum force in cells treated with the RAR-β agonist CD 2314 compared to vehicle control (Supplementary Figure S2). Together these results indicate that RAR-β activation decreases cell contractility via MLC-2 downregulation, consistent with the role of the latter as a regulator of actomyosin contractility.

Cytoskeletal stiffness is another indicator of biomechanical activity that depends on the regulation of the actomyosin cytoskeleton. The ability to dynamically reorganise the cytoskeleton in response to the changing microenvironment is critical in cancer cell migration and correlates with invasive potential [54]. We analysed cytoskeletal stiffness using atomic force microscopy (AFM) to carry out nanoindentation measurements of individual cells (Fig. 3D). Control Suit2 cells showed a mean stiffness of 292 ± 20 Pa (mean ± SEM, *n* = 91 cells), comparable to similar cell types, whereas cells treated with RAR-β agonist for 72 h showed reduced cortical stiffness (202 ± 12 Pa, mean ± SEM, *n* = 58 cells), consistent with the decrease in MLC-2 expression (Fig. 3E). We also observed that both knockdown of the RAR-β receptor (RAR-β siRNA) or MLC-2 overexpression recovered control-level of cytoskeletal stiffness. These results indicate that RAR-β activation causes an MLC-2 dependent reduction in cortical stiffness.

Mechanosensing is the ability for cells to sense and respond to mechanical cues. Like traction force generation, mechanosensing necessitates an intact actomyosin machinery that can dynamically reorganise in response to mechanical stimuli. Mechanical signals, including substrate stiffness, play an important role in directing cancer cell migration and invasion and are therefore one of the driving forces behind pancreatic cancer progression. Here we assessed the effect of RAR-β signalling on mechanosensing using magnetic tweezers (Fig. 3F). Suit2 cells were incubated with fibronectin-coated magnetic beads, which readily attach to surface integrins, and subjected to a pulsatile force regime (12 force pulses, 6 nN, 3 s per pulse), while the resulting bead displacements over the 12 pulses were monitored to measure cell stiffening in response to force application.

Control Suit2 cells showed significant cytoskeletal reinforcement, with a decrease in relative bead displacement between the 1st and 12th pulse (~40%), indicative of their mechanosensing capacity (Fig. 3G, H). Conversely, cells treated with RAR-β agonist for 24 h showed a decrease in cytoskeletal reinforcement, with only a ~ 15% reduction in the amplitude of the 12th pulse relative to the 1st pulse, and a significantly larger bead displacement on the 12th pulse (0.84 ± 0.06, mean ± s.e.m, *n* = 22) compared to control cells (0.63 ± 0.02. mean ± s.e.m, *n* = 41, *p* < 0.001 Dunnett’s multiple comparisons test), indicative of impaired mechanosensing. Consistent with our previous findings, knockdown of RAR-β via siRNA inhibited the effect of the agonist on mechanosensing, and overexpression of MLC-2 restored the mechanosensing capacity of Suit2 cells to control levels indicating that RAR-β modulates mechanosensing in PDAC cells in an MLC-2 dependent manner.

### RAR-β activation impairs cancer cell invasion

The first step in the metastatic journey is the breaching of the basement membrane (BM), a complex sheet-like protein bilayer that provides anchoring for the basal surface of epithelial cells and promotes apico-basal polarity. The process of epithelial-to-mesenchymal transition that accompanies cancer progression is characterised by a loss of cell polarity, cell-cell junctions, and an increase in cell mobility. We recently reported that the basement membrane of PDAC differs in structure and composition from that of the healthy pancreas [53], resulting in an abnormal mechanical interaction between cancer cells and the basement membrane that promotes the breaching of the basement membrane or transmigration.

The ability to breach the initial barrier posed by the basement membrane enables tumour cells to invade neighbouring tissues and is therefore a key marker of malignancy and a critical therapeutic target in the prevention of metastasis. In order to investigate the effect of RAR-β activation on the invasive ability of cancer cells, we used a recently developed BM mimic based on mouse mesenteries [37]. Mouse mesenteries present a composition and bilayer structure similar to PDAC basement membranes and are therefore ideal models to study BM transmigration.

**Fig. 4:**
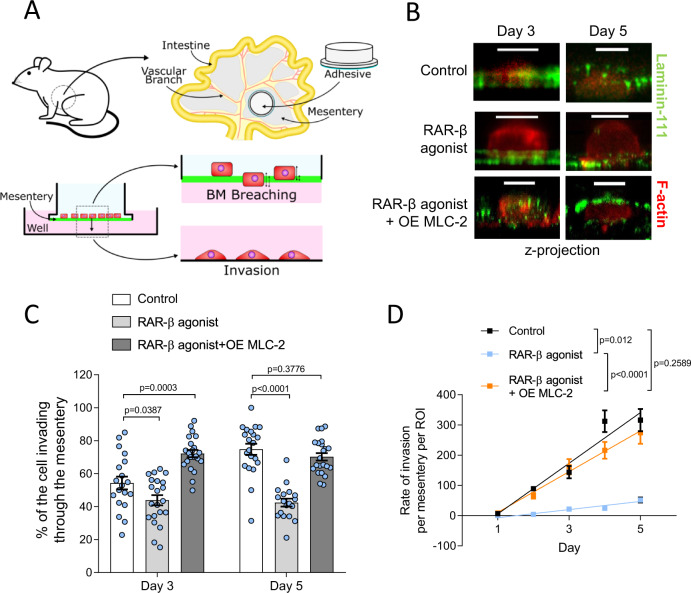
RAR-β activation reduces cell invasion. **A** Schematic of the mesentery preparation and invasion assay. Mesenteries from wild type mice are surgically extracted and bonded to hollow cylindrical tubes. After decellularisation, Suit2 cells are seeded on the mesentery transwells and cultured for up to 5 days. Every 24 h, mesenteries are transferred to a new well, and the number of cells attached to the well (complete migration) are counted. Mesenteries are fixed on days 3 and 5 to quantify percentage invasion. **B** Confocal fluorescence images (z-projection) of Suit2 cells in control, RAR-β agonist, and RAR-β agonist + MLC-2 overexpression (OE MLC-2) conditions invading through mesenteries. Laminin-111 (green), f-actin (red). Scale bar: 10 μm. **C** Quantification of the invasive capacity of Suit2 cells. The percentage of the cell body that had invaded through the bilayer was quantified at day 3 (mean ± s.e.m., *n* = 19, 21 and 22 cells for control, RAR-β agonist and RAR-β agonist + MLC-2 overexpression (OE MLC-2), respectively) and day 5 (mean ± s.e.m., *n* = 21, 18 and 22 cells for control, RAR-β agonist and RAR-β agonist + MLC-2 overexpression (OE MLC-2), respectively). *P*-values indicate significant difference relative to control by one way ANOVA test with Dunnett’s post-hoc test for Day 3 and Day 5. **D** Cumulative number of cells that have fully invaded through the membrane over a period of 5 days for Suit2 control, RAR-β agonist and RAR-β agonist + MLC-2 overexpression (OE MLC-2). Mean ± s.e.m., *n* = 28, 18, 26 (day 1), 53, 40, 48 (day 2), 67, 47, 56 (day 3), 71, 49, 60 (day 4), 78, 65, 68 (day 5). Lines represent linear regression model, with *p*-values indicating significant difference between the slopes of the linear regression.

Mouse mesentery models were isolated and prepared as described by Ghose et al. [37]. (Fig. 4A). Suit2 cells were then cultured on the decellularised mesenteries and their transmigration across the bilayer was monitored over a period of 5 days using confocal fluorescence microscopy (Fig. 4B). We characterised the percentage of the cell body that penetrated the bilayer structure on days 3 and 5 (Fig. 4C) and found that in control Suit2 cells, the percentage of the cell body invading through the mesentery increased from 54 ± 4% at day 3 to 75 ± 3% at day 5 (mean ± s.e.m, *n* = 19 and 21 cells, respectively). Conversely, cells treated with RAR-β agonist showed no increase in invasion between days 3 (44 ± 3%) and 5 (42 ± 2%, mean ± s.e.m, *n* = 21 and 18 cells, respectively) and a significantly lower percentage of invasion compared to control cells at both day 3 and day 5. Overexpression of MLC-2 restored the invasive potential of RAR-β agonist-treated cells, showing a high invasive potential both at day 3 (72 ± 2%) and day 5 (70 ± 2%, mean ± s.e.m, *n* = 22 cells). Measurement of cumulative invasion, i.e., the number of cells that fully migrated through the mesentery (per ROI) showed a similar trend (Fig. 4D), with RAR-β treated cells showing decreased invasive potential over the 5-day period compared to control Suit2 cells and Suit2 cells overexpressing MLC-2.

## Discussion

Retinoids, the active forms of vitamin A, are a family of compounds with pleiotropic effects on cells that act through the ligand-activated transcription factors of the retinoic acid receptor (RAR) family. Loss of RAR expression, particularly RAR-β, is associated with a variety of cancers [29, 38], which prompted their use in cancer treatment, particularly for acute promyelocytic leukaemia (APL) [31]. Here, we report that RAR-β expression is reduced in pancreatic ductal adenocarcinoma (PDAC) and its downregulation correlates with tumour stage, pointing towards RAR-β as an interesting target in PDAC.

**Fig. 5:**
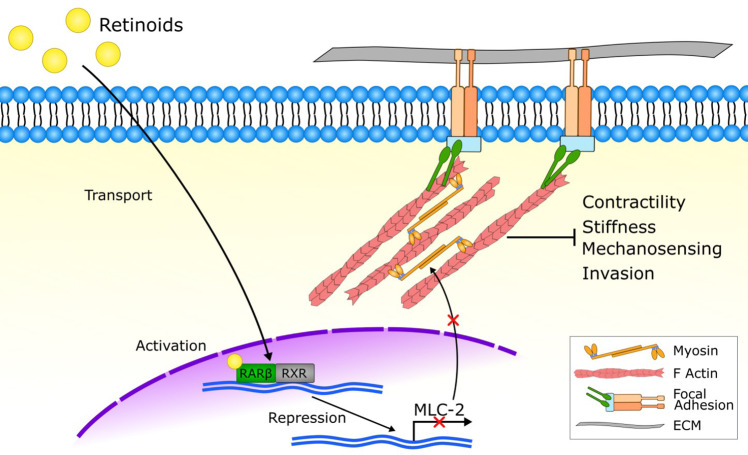
RAR-β modulates mechanical activity in PDAC cells via MLC-2. Retinoids activate the nuclear retinoid acid receptor β (RAR-β) along with its transcription partner RXR, which downregulate the expression of myosin regulatory light chain 2 (MLC-2), in turn decreasing actomyosin activity. By targeting the cytoskeleton, retinoids modulate traction force generation, mechanosensing, cortical stiffness and basement membrane invasion.

Retinoids have been previously shown to inhibit cell growth and induce apoptosis in several PDAC cell lines alone or in combination with gemcitabine [55–57]. Here we found that, when activated by retinoids, RAR-β downregulates the expression of MLC-2, in turn modulating actomyosin contractility, stress fibre formation and cortical stiffness. Force generation by actomyosin governs the ability for cells to mechanosense their substrate, and is a key driver in mesenchymal and amoeboid migration in cancer [58] (Fig. 5). These results position RAR-β as a regulator of cancer biomechanics, building on its previously established role as a cell growth inhibitor and an attractive therapeutic target in cancer.

Despite the potential of retinoids as anticancer drugs, their clinical application has been limited by tumour chemoresistance. Resistance to retinoids has been observed in certain PDAC cell lines, as well as other types of cancer, and may be associated with a deficiency in the cellular retinoic acid-binding protein 2 (CRABP2) [55, 59], which is involved in the intracellular transport of retinoic acid [60–62]. The loss of RAR-β expression has also been proposed as a mechanism of chemoresistance, which is often attributed to epigenetic (methylation) changes in the RAR-β promoter, leading to its silencing [55, 59]. In this context, the positive autoregulation by RAR-β signalling that we have observed in PDAC cells could be essential to the success of retinoids by increasing PDAC sensitivity to treatment. Future work will be required to investigate the mechanisms of transport and bioavailability of retinoids in order to design effective therapeutic strategies.

More importantly, despite research efforts, the predictive markers of resistance and response to retinoids remain elusive. This is particularly important in light of the potential adverse effects of retinoid treatment. For instance, the RAR-β isoform RAR-β_4_ has been associated with oesophageal carcinoma [63], while the paralog RAR-γ promotes tumorigenesis in PDAC and is associated with a poor prognosis [64], pointing towards the need for therapeutic strategies that are highly specific. In addition, several retinoic acid transport proteins have been found to correlate with increase pancreatic cancer cell motility and invasion, including CRABP2 [65] and FABP5 [62], while CRABP1 correlates with worse prognosis in breast cancer [66]. These findings highlight the need for a comprehensive library of predictive markers of retinoid response in order to develop accurate patient stratification strategies.

In recent years, the role of mechanical cues, such as tissue stiffness, in the progression of cancer has come into focus [4, 67]. Cancer associated fibroblasts (CAFs), such as PSCs develop and maintain the aberrant microenvironment that drives PDAC progression, characterised by excessive extracellular matrix (ECM) deposition, remodelling and stiffness. Anti-stromal therapies that aim to deplete the tumour ECM, however, have proven to be unsuccessful, in some instances resulting in more aggressive tumours [68, 69]. The role of the stroma in tumour progression is complex and changes along cancer’s spatiotemporal evolution, prompting the development of novel therapies that modulate or reprogramme the crosstalk between cancer cells and CAFs [70–72]. Our group and others have previously shown that retinoids can mechanically reprogramme PSCs to inhibit matrix remodelling [11, 73–75, 76]. Retinoid treatment could therefore act synergistically to modulate the mechanical activity of both cancer cells and CAFs, directly targeting the mechanical interaction between cancer cells and their microenvironment.

Here we have observed that retinoid treatment decreases the invasive potential of PDAC cells, pointing towards a potential role in the prevention of metastasis. Metastasis is a complex, multi-stage process that involves cancer cell migration, intravasation and colonisation of distant tissues, which rely on rapid actomyosin reorganisation [77]. Cell contractility also regulates the secretion of matrix metalloproteinases [12], which are required to remodel the ECM. Moreover, retinoids can regulate the mechanical activity of hepatic stellate cells, which may hinder the development of the premetastatic niche in the liver, the primary metastatic site for PDAC [25, 78]. These findings suggest that targeting the RAR-β/MLC-2 axis could be a compelling strategy to impair cancer metastasis, and positions this pathway as an important player in therapies aimed at mechanically modulating the tumour and its microenvironment. Future studies using animal models will be required to elucidate the systemic effects of retinoids and to understand the interaction between their mechano-modulating and their anti-proliferative effects.

## Supplementary information

Supplementary methods and figures

Supplementary data 1

Supplementary data 2

## Data Availability

High-throughput sequencing data that support the findings of this study have been deposited to ArrayExpress (https://www.ebi.ac.uk/biostudies/arrayexpress) under accession number E-MTAB-12792. The data that support the findings of this study and the custom MATLAB codes are available from the corresponding authors upon reasonable request.
