# Supplementary material for: Retinoic acid receptor β modulates mechanosensing and invasion in pancreatic cancer cells via myosin light chain 2
Source: Oncogenesis. 2023 May 2;12(1):23. doi: 10.1038/s41389-023-00467-1 (PMC10154384; doi:10.1038/s41389-023-00467-1)
Supplement: Supplementary file 1 — Supplementary methods and figures [file 41389_2023_467_MOESM1_ESM.pdf]

Retinoic acid receptor β modulates mechanosensing and invasion in pancreatic cancer cells via myosin light chain 2

Carlos Matellan[1‡], Dariusz Lachowski[1‡], Ernesto Cortes[2‡], Kai Ning Chiam[3], Aleksandar Krstic[3,4], Stephen D. Thorpe[3,5,6,*], and Armando E. del Río Hernández[1,*]

1 Cellular and Molecular Biomechanics Laboratory, Department of Bioengineering, Imperial College London, London SW7 2AZ, UK

2 Department of Physiology, School of Medicine, Autonomous University of Madrid, 28029 Madrid, Spain

3 UCD School of Medicine, University College Dublin, Dublin, Ireland

4 Systems Biology Ireland, University College Dublin, Dublin, Ireland

5 UCD Conway Institute of Biomolecular & Biomedical Research, University College Dublin, Dublin, Ireland

6 Trinity Centre for Biomedical Engineering, Trinity College Dublin, Dublin, Ireland

[‡] These authors contributed equally to this work

*Corresponding author e-mails: a.del-rio-hernandez@imperial.ac.uk, stephen.thorpe@ucd.ie

This document includes

Description of supplementary data files

Supplementary Materials and Methods

Supplementary Figures S1 to S5

Supplementary Figure Captions

References for Supplementary Information

Supplementary Data files

Supplementary Data 1: ChIP-seq peak positions and associated genes identified after treatment with RAR-β agonist relative to RAR-β antagonist.

Supplementary Data 2: STRING and Ingenuity Pathway Analysis terms and summaries.

Supplementary Materials & Methods

1) Antibodies and Immunostaining

Suit2 cells were seeded on coverslips previously treated with Fibronectin (Gibco, PHE0023) for 45 minutes and washed with D-PBS (Sigma-Aldrich, D8537). Cells were fixed with 4% paraformaldehyde (Sigma-Aldrich, P6148) diluted in PBS for 10 min. Fixed cells were permeabilised with 0.5% saponin (Sigma-Aldrich, 47036) diluted in PBS for 10 min. Cells were then incubated in a blocking solution of 1% bovine serum albumin (BSA; Sigma-Aldrich, A8022, Lot SLBT9577) and 22.52 mg/mL UltraPure Glycine (Invitrogen, Lot 18C2856902) diluted in PBS with 0.1% Tween 20 (Sigma-Aldrich, P1379) for 30 min. Cells on coverslips were incubated with diluted primary antibodies in PBST against myosin light chain-2 (MLC-2, Cell Signaling Technology, 3672S, Rabbit, 1:100), RAR-β (Abcam, ab53161, Rabbit, 1:50) and YAP (Cell Signaling Technology 4912S, Rabbit, 1:50) at 4 °C overnight. This was followed by incubation with goat anti-rabbit 488 (Life Technologies, A11034, 1:200) secondary antibody and Phalloidin-594 (Abcam, ab176757, 1:200) for 1 h at room temperature. Cells were then mounted with Prolong Gold antifade reagent containing DAPI (ThermoFisher Scientific) and imaged on a Nikon Eclipse Ti-E microscope with a 40× objective (Nikon, Kingston-upon-Thames UK). Fluorescence intensity was quantified in Fiji [1].

Fixed Suit2 cells on mesenteries were permeabilised using 0.5% Triton X-100 (Sigma-Aldrich, T8787). Fixed samples were blocked using 2% BSA. PBS was used to block non-specific binding for 30 min. Samples were then incubated with primary antibodies for laminin (Rabbit, Sigma-Aldrich, L9393, 1:100) at room temperature for 1 h. This was followed by incubation with goat anti-rabbit Alexa Fluor 488 (ThermoFisher, A11034, 1:200) secondary antibody and Phalloidin-594 (Abcam, Ab176757, 1:200 dilution) for 1 hour at room temperature. Samples were then mounted using Prolong Gold antifade reagent with DAPI (Thermo Fisher Scientific) and imaged using confocal microscopy with a Nikon Eclipse Ti-E microscope, 60× objective (Nikon, Kingston-upon-Thames UK). 3D confocal images were acquired with 0.25 µm z-section spacing.

2) Tissue microarray staining

For quantification of the levels of RAR-β and MLC-2 in human pancreatic tissues from healthy, cancer adjacent and PDAC tissues, tissue micro arrays (TMAs) were obtained from Biomax (Catalogue number PA803 for RAR-β, PA242e for MLC-2). Formalin fixed, paraffin embedded pancreatic ductal adenocarcinoma and normal tissue arrays (US Biomax inc., cat. PA803) were dewaxed in histoclear (National Diagnostics, cat. HS-200) and rehydrated in decreasing concentrations of ethanol. Subsequently, samples underwent heat induced epitope retrieval in pH 6.0 citrate buffer for 30 minutes at 95°C. After cooling to room temperature, the array was washed in tris-buffered saline (TBS) plus 0.025% Triton X-100 (Sigma, T8787) and blocked in 10% normal goat serum (Sigma-Aldrich) with

1% BSA in TBS for 2 h at room temperature. Blocked samples were incubated overnight at 4°C with anti-RAR-β antibodies (Abcam, ab53161, Rabbit, 1:100) or anti-MLC-2 antibodies (Cell Signaling Technology, 3672S, Rabbit, 1:100) and anti-PAN cytokeratin (Abcam, ab6401, 1:250) in TBS with 1% BSA. Following the incubation, samples were washed in TBS plus 0.025% Triton X-100 with gentle agitation and incubated with secondary antibodies, goat anti-mouse Alexa Fluor 488 (ThermoFisher, A-11030) and goat anti-rabbit Alexa Fluor 546 (ThermoFisher, A-11035) diluted 1:400 in TBS with 1% BSA for 1 hour at room temperature. Tissue microarrays were mounted using Prolong Gold antifade reagent with DAPI (ThermoFisher Scientific) and imaged with a Nikon Eclipse Ti-E microscope, 20x objective (Nikon, Kingston-upon-Thames UK). Mean fluorescence intensity of the images was quantified using Fiji [1].

3) Immunofluorescence Image Analysis

Widefield fluorescent images were taken with an inverted microscope (Nikon Ti Eclipse, C-LHGFI HG Lamp, CFI Plan Fluor 40× NA 0.6 air objective; Nikon Europe, Amsterdam, Netherlands) fitted with a Neo sCMOS camera (Andor, Belfast, UK) with NIS elements AR software. Staining fluorescence intensity was quantified in Fiji using the “mean grey value” parameter applied to a region of interest (ROI). Regions of interest (ROI) for individual cells were segmented based on their actin staining (red channel). For YAP nuclear localisation, nuclear ROIs were defined through automated thresholding of the DAPI (nuclear) channel, and cytoplasmic ROIs were defined as the whole cell ROI with subtracted nuclear ROI. Ratios of the nuclear to cytoplasm fluorescence intensities (“mean grey value”) were calculated in order to analyse the localization of YAP in the different cells. Unless otherwise specified, individual data points represent the average intensity (or average nuclear/cytoplasmic intensity ratio) for each field of view (10-20 cells quantified per field of view) across three independent replicates.

4) qPCR

Total RNA was extracted using the RNeasy Mini kit (Qiagen, 74104) and 1 µg of total RNA was reverse-transcribed using the High-Capacity RNA-to-cDNA kit (Applied Biosystems, 4387406) according to the manufacturer’s instructions. Real-time quantitative PCR (qPCR) was performed using the SYBR Green PCR Master Mix (Applied Biosystems, 4309155). Primer sequences: RPLP0: forward 5'-CGGTTTCTGATTGGCTAC-3' and reverse 5'-ACGATGTCACTTCCACG-3'; RAR-β: forward 5’-AATAAAGTCACCAGGAATCG -3’ and reverse, 5’- CAGATTCTTTGGACATTCCC -3’; MLC-2: forward, 5’-ATCCACCTCCATCTTCTT-3’ and reverse, 5’-AATACACGACCTCCTGTT-3’. RT qPCR data was analysed using the $2^{-\Delta\Delta CT}$ method with RPLP0 as endogenous control gene. Error bars for RT qPCR data were calculated as $2^{-(\Delta\Delta CT \pm SEM)}$ as described in [2].

5) Identification of putative RAREs

Retinoic Acid Response Element (RARE) binding site prediction was performed for MYL, MYL9 and MYL10 promoters. Analysis was focused on 1,000 bp upstream of the corresponding transcription start site (TSS) for each gene. Prediction of putative binding sites was performed using the LASAGNA tool [3]. The following analysis parameters were used: Transcription factor (TF) model input was based on TRANSFAC database of TF binding sites with a focus on RXR:RAR DR5 (Matrix-derived JASPAR CORE models in Vertebrates) and RAR-β (LASAGNA-aligned TRANSFAC models in Homo sapiens). Promoter sequences were fetched by the tool, with transcription factor binding site (TFBS) prediction analysis performed on 1,000 bp upstream of the respective TSS. The cut-off *p*-value was set to 0.001. Chromosomal locations for putative RAREs were remapped to the hg38 assembly using NCBI Genome Remapping Service (https://www.ncbi.nlm.nih.gov/genome/tools/remap).

6) Chromatin immunoprecipitation sequencing and analysis

ChIP-seq was performed on Suit2 cells treated for 72 h with either DMSO (vehicle control), 1 µM RAR-β agonist (CD 2314, Tocris 3824), or 1 µM RAR-β antagonist (LE 135, Tocris 2021). Chromatin was immunoprecipitated using the SimpleChIP Plus Sonication Chromatin IP kit (Cell Signaling Technology, 56383) according to manufacturer's instructions using a rabbit polyclonal anti RAR-β antibody (Abcam ab53161) at 2 µg sample. Rabbit monoclonal anti-histone H3 (clone D2B12, Cell Signaling Technology, 4620) was used as a positive control. ChIP-seq libraries were prepared from NEBNext Ultra II DNA library Prep Kit (Illumina) and were sequenced on an Illumina NextSeq 500 with paired-end 75-bp reads for n = 3 biological replicate samples. Reads were trimmed using Trim Galore! (https://www.bioinformatics.babraham.ac.uk/projects/trim_galore/). ChIP-seq data was aligned to the hg38 genome assembly using Bowtie2 [4]. To increase read depth and improve peak finding, data from three biological replicates was combined using Merge BAM Files (version 1.1.2). ChIP-seq peak detection and visualisation was performed using EaSeq (http://easeq.net) [5]. Regions were identified in the agonist condition using the antagonist condition as a negative control with global thresholding and a window size of 300 bp.

String (version 11.0, www.string-db.org) was used to generate protein-protein interaction networks, and for gene ontology (GO) and Reactome pathway analysis. Ingenuity Pathway Analysis (IPA) software (Qiagen) was also used for pathway analysis. Bubbleplots were plotted using http://www.bioinformatics.com.cn/srplot.

Supplementary Figures

A

MYL2 (NM_000432), TSS - chr12:110,920,600 strand -						
RA binding element	*Sequence*	*Position relative to TSS*	*Strand*	*Score*	*p-value*	*E-value*
RAR-beta (T00721)	CTGCCAAAAGCGGTCATGGGGT	947	+	145.64	0.000975	0.95
**MYL9 (NM_006097), TSS – chr20:36,541,484 strand +**						
RA binding element	*Sequence*	*Position relative to TSS*	*Strand*	*Score*	*p-value*	*E-value*
RAR-beta (T00721)	CGGCAGCAGGAGGTCAGGAGG	319	-	150.83	0.0006	0.59
RAR-beta (T00721)	CGGCAGCAGGAGGTCAGGAG	320	-	146.61	0.000925	0.9
RAR-beta (T00721)	CGGCAGCAGGAGGTCAGGAGGA	318	-	145.71	0.000975	0.95
**MYL10 (NM_138403), TSS – chr7:101629296 strand -**						
RA binding element	*Sequence*	*Position relative to TSS*	*Strand*	*Score*	*p-value*	*E-value*
RXR::RAR_DR5 (MA0159.1)	GGTGCAATCACAGCTCA	207	+	9.54	0.000225	0.221

B

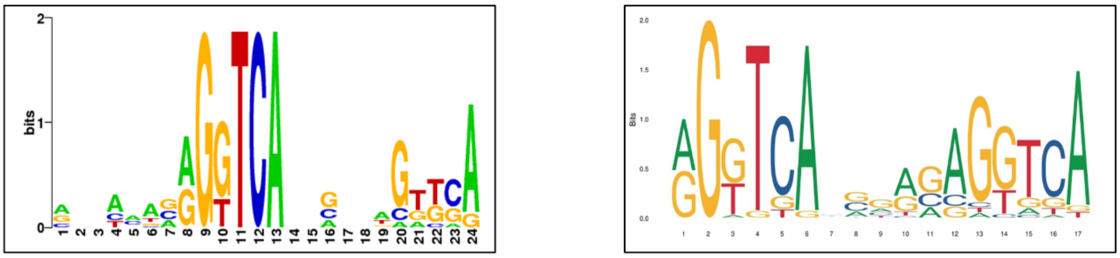

RAR-beta (T00721) RXR::RAR_DR5 (MA0159.1)

Supplementary Figure S1. Identification of putative RAREs. (A) Putative retinoic acid reactive elements (RAREs) sites identified with the LASAGNA tool within the 1,000 bp region upstream of the transcription start site for three myosin light chain 2 isoforms (MYL2, MYL9 and MYL10). **(B)** Consensus RARE sequence logos (with TRANSFAC and JASPAR database ID) for RAR-beta and RXR:RAR DR5, respectively.

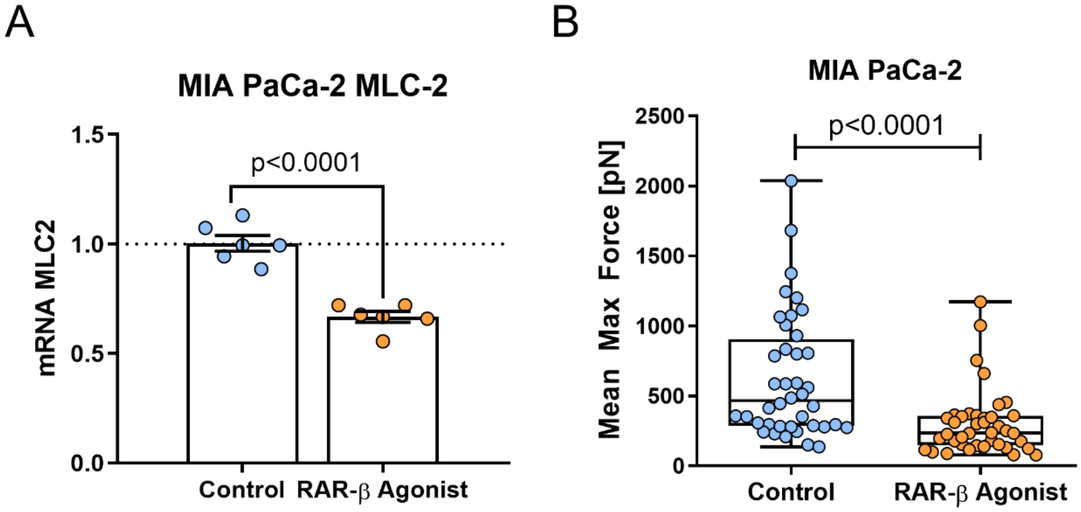

Supplementary Figure S2. RAR-β regulates MLC-2 expression and traction force generation in MIA PaCa-2 cells. (**A**) Relative mRNA expression of MLC2 in Vehicle Control and RAR-β agonist (CD 2314) treated MIA PaCa-2 cells measured by RT qPCR. mRNA expression normalised to RPLP0. Mean ± s.e.m., *n* = 6. P-value denote significant difference by unpaired t-test. **(B)** Quantification of mean maximum traction force exerted by MIA PaCa-2 cells on elastic pillars. *n* = 40 cells for vehicle control (DMSO) and RAR-β agonist (CD 2314) treated cells. P-value denote significant difference by Mann-Whitney test.

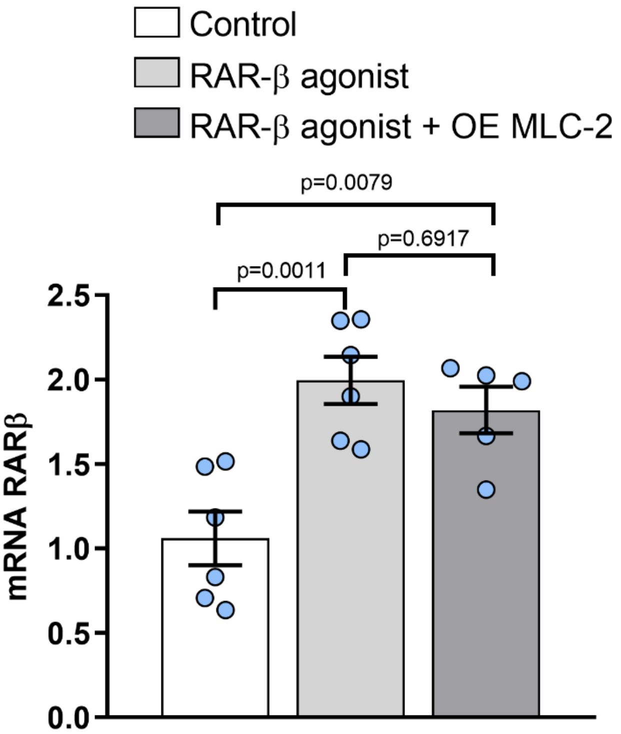

Supplementary Figure S3. MLC-2 overexpression does not affect RAR-β expression. Relative expression of RAR-β in Control, RAR-β agonist, RAR-β agonist + MLC-2 overexpression respectively as measured by mRNA RT qPCR normalised to RPLP0. Control and RAR-β agonist data reproduced from Figure 1F. Geometric mean ± s.e.m, *n* = 6, 6, 5. P-value indicates significant difference by one way ANOVA test with Tukey's post-hoc test.

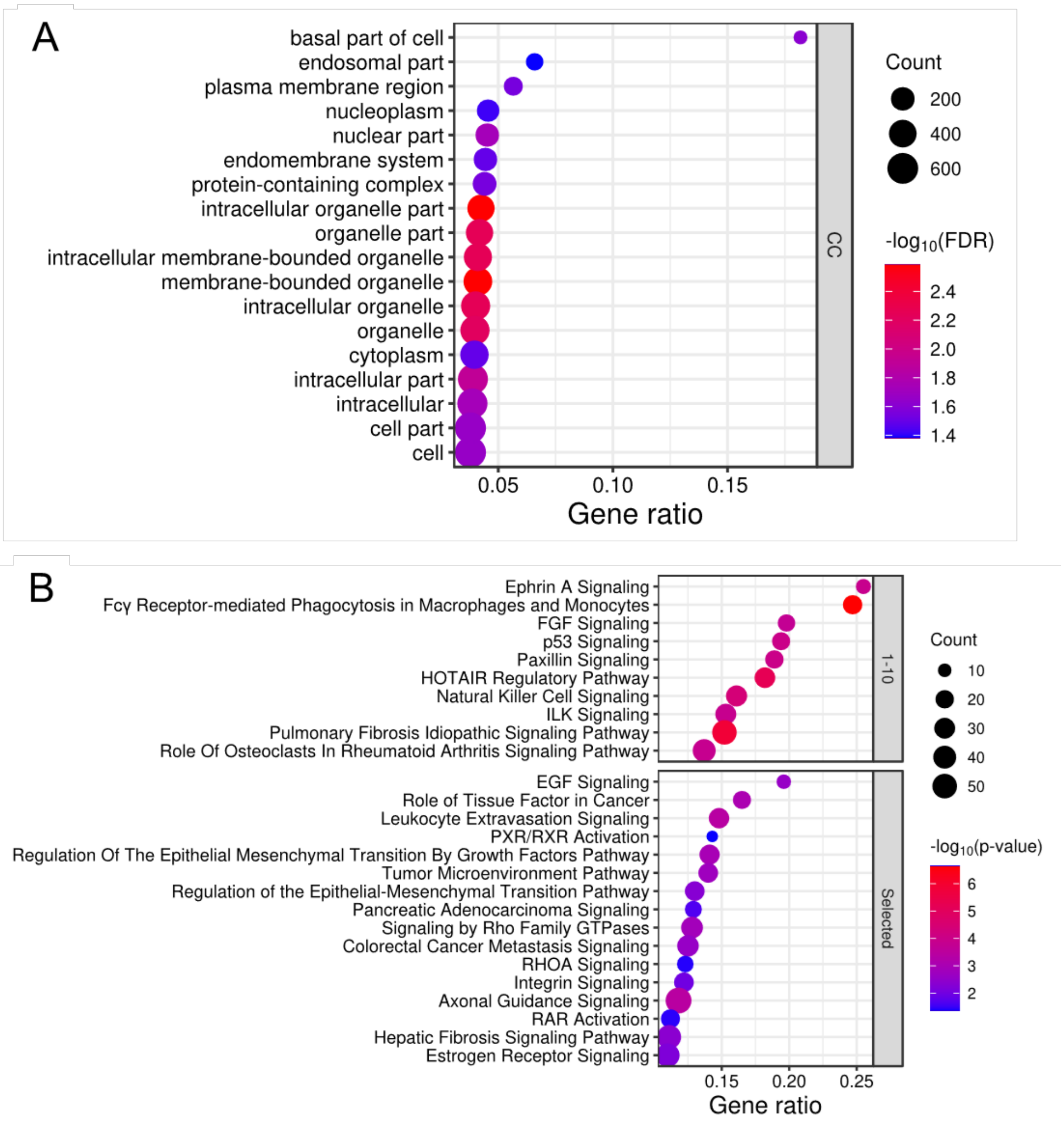

Supplementary Figure S4. RAR-β ChIP-seq identifies gene ontology terms and signalling pathways associated with cell contractility. (A) Significant gene ontology terms for cellular component identified using RAR-β enriched genes with a $\log_2$(fold enrichment) > 1.5 in STRING. **(B)** Canonical pathways identified using Ingenuity Pathway Analysis on the full list of RAR-β enriched genes with p-value included in the analysis. The top 10 pathways are provided alongside 16 pathways with relevance to cell contractility, migration, and cancer progression. The presence of PXR/RXR Activation and RAR Activation in this list indicates that RAR-β agonist treatment was effective.

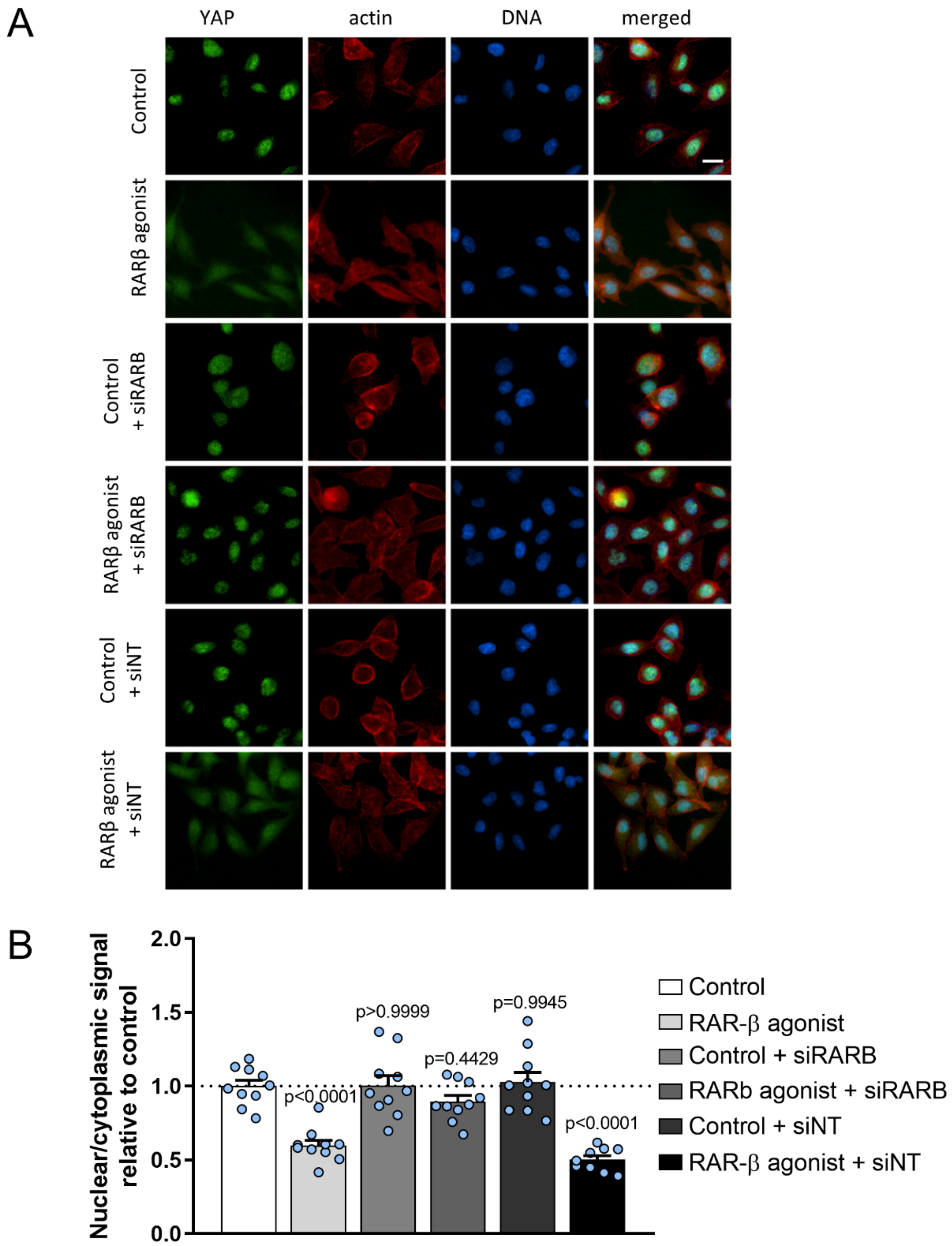

Supplementary Figure S5. RAR-β activation induces mechanical quiescence in Suit-2 cells. (A) Representative images of immunofluorescence analysis of YAP-1 localisation within the cell for control, RAR-β agonist, RAR-β siRNA, RAR-β agonist + RAR-β siRNA, non-targeting siRNA (siNT), and RAR-β agonist + siNT. YAP (green), f-actin (red), DNA (blue). Scale bar: 20 µm. **(B)** Quantification of the nuclear/cytoplasmic ratio of YAP-1 location based on the staining in (A). Mean ± s.e.m., *n* = 10 fields of view for control, RAR-β agonist, RAR-β siRNA, RAR-β agonist + RAR-β siRNA, and siNT, *n* = 9 fields of view for RAR-β agonist + siNT respectively. P-values indicate significant difference relative to control by one way ANOVA test with Dunnett's post-hoc test.

References (Supplementary Methods)

1 Schindelin J, Arganda-Carreras I, Frise E, Kaynig V, Longair M, Pietzsch T et al. Fiji: an open-source platform for biological-image analysis. Nature Methods. 2012; 9:676-682.

2 Taylor SC, Nadeau K, Abbasi M, Lachance C, Nguyen M, Fenrich J. The Ultimate qPCR Experiment: Producing Publication Quality, Reproducible Data the First Time. Trends in Biotechnology. 2019; 37:761-774.

3 Lee C, Huang C-H. LASAGNA: A novel algorithm for transcription factor binding site alignment. BMC Bioinformatics. 2013; 14:108.

4 Langmead B, Salzberg SL. Fast gapped-read alignment with Bowtie 2. Nat Methods. 2012; 9:357-359.

5 Lerdrup M, Johansen JV, Agrawal-Singh S, Hansen K. An interactive environment for agile analysis and visualization of ChIP-sequencing data. Nat Struct Mol Biol. 2016; 23:349-357.
